# Efficacy and safety profile of combining antiangiogenic agents with chemotherapy in patients with advanced malignant pleural mesothelioma: A systematic review and meta-analysis of randomized controlled trials

**DOI:** 10.1371/journal.pone.0295745

**Published:** 2023-12-21

**Authors:** Wei Tian, Qian Guo, Daidi Fu, Xiao Ma, Rui Wang

**Affiliations:** 1 Department of Oncology, Zibo Central Hospital, Shandong University, Zibo, Shandong, Peoples’ Republic of China; 2 Day Care Unit, Zibo Central Hospital, Shandong University, Zibo, Shandong, Peoples’ Republic of China; 3 Department of Internal Medicine, Zhangqiu People’s Hospital, Zhangqiu, Shandong, People’ Republic of China; Inha University Hospital, REPUBLIC OF KOREA

## Abstract

**Objectives:**

Several prospective trials had been reported on chemotherapy with or without antiangiogenic agents in patients with advanced malignant pleural mesothelioma (MPM), with diverse results. We performed this systematic review and meta-analysis to evaluate the efficacy and safety of the combination regimen.

**Methods:**

We systematically identified trials in several databases, including MEDLINE, EMBASE, Cochrane Central Register of Controlled Trials, ASCO Abstracts and ESMO Abstracts. All the randomized controlled trials (RCTs) about chemotherapy combined with antiangiogenic agents in advanced MPM were identified. Overall survival (OS) was the primary outcome, while progression-free survival (PFS), overall response rate (ORR) and serious toxicities were the secondary outcomes. Review Manager 5.3 was used to perform the statistical analyses. Stata 12.0 was used to assess the publication bias of egger’s test.

**Results:**

5 randomized controlled trials containing 1250 patients were finally included in this analysis. Statistical analyses showed that the addition of antiangiogenic agents to chemotherapy could prolong OS [HR 0.79 (0.71–0.89), p<0.0001] and PFS [HR 0.75 (0.68–0.84), p<0.00001] in advanced MPM, especially in the epithelioid subgroup, with a tolerable toxicity profile. No significant difference was found in the analysis of ORR [HR 1.13 (0.95–1.35), p = 0.18]. Heterogeneity was found in the analyses of PFS and ORR, which might be caused by the limitation in uniform evaluation of tumor response.

**Conclusions:**

The combination of antiangiogenic agents with chemotherapy showed superior over chemotherapy alone in patients with advanced MPM. More prospective trials should be warranted to identify patients who would most likely benefit from the combination regimen.

## Introduction

Mesothelioma is a rare but aggressive cancer, with 30,870 new cases and 26,278 deaths worldwide in 2020 [1]. Malignant pleural mesothelioma (MPM), the most common form of malignant mesothelioma, is usually diagnosed at the advanced stage. MPM is classified into 3 main histologic subtypes: epithelioid, sarcomatoid and biphasic [2]. For patients with unresectable disease, chemotherapy of pemetrexed combined with platinum is the basic treatment option [3]. Although regimen of pemetrexed plus cisplatin brings clinical benefits compared with cisplatin alone, the median overall survival (OS) is only 12.1 months [4].

Angiogenesis plays an important role in cancer progression, which has become an important target in cancer therapy [5]. Vascular endothelial growth factor (VEGF), fibroblast growth factor (FGF), platelet-derived growth factor (PDGF) and their corresponding receptors regulate the signaling pathways of cancer angiogenesis. Signaling pathways involved in angiogenesis are thought to be associated with the development of MPM [2]. The VEGF serum levels are verified to be higher in patients with MPM [6]. VEGF receptor 2, FGF receptor 1, FGF2, and FGF18 have been found to be overexpressed in mesothelioma cells [7, 8]. PDGF also plays a role as autocrine growth stimulating factor in the development of malignant mesothelioma [9]. Several studies have investigated antiangiogenic agents such as cediranib, sorafenib and sunitinib in the second-line setting when used as monotherapy in advanced MPM. However, the results did not demonstrate sufficient clinical benefits in these studies [10–12].

In the Mesothelioma Avastin Cisplatin Study, bevacizumab combined with pemetrexed and cisplatin significantly improved the OS compared with pemetrexed and cisplatin alone, which indicated that addition of antiangiogenic agents to standard chemotherapy might be a rational approach in MPM [13]. Over the past several years, a number of randomized controlled trials (RCTs) have evaluated the efficacy of adding antiangiogenic agents to standard chemotherapy in patients with advanced MPM, but with diverse results. Therefore, we performed this systematic review and meta-analysis to compare the antiangiogenic agents plus standard chemotherapy versus chemotherapy alone for such patients.

## Materials and methods

### Search strategy

We collected all the relevant studies by searching MEDLINE, EMBASE, Cochrane Central Register of Controlled Trials, ASCO Abstracts and ESMO Abstracts up to June 2023. In order to identify all the RCTs in MEDLINE (Ovid format), the Cochrane highly sensitive search strategy was used. **Table 1** showed the search strategy used in this analysis.

**Table 1:** Search strategy for MEDLINE (Ovid format) used in this meta-analysis.

1. randomized controlled trial.pt.	18. antineoplastic$.tw.
2. controlled clinical trial.pt.	19. or/15-18
3. randomized.ab.	20. aflibercept
4. placebo.ab.	21. sorafenib
5. drug therapy.fs.	22. sunitinib
6. randomly.ab.	23. axitinib
7. trial.ab.	24. apatinib
8. groups.ab.	25. vandetanib
9. or/1-8	26. nintedanib
10. humans.sh.	27. pazopanib
11. 9 and 10	28. cediranib
12. exp pleural mesothelioma/	29. bevacizumab
13. (pleural adj5 mesothelioma$).mp.	30. ramcirumab
14. or/12-13	31. or/20-30
15. exp drug therapy/	32. 14 and 19
16. chemothera$.tw.	33. 31 and 32
17. drug therap$.tw.	34. 11 and 33

We identified all the randomized controlled trials about chemotherapy combined with angiogenesis agents in advanced MPM. All references of the included studies were scanned manually to identify relevant trials. There was no language restriction in the search.

### Inclusion and exclusion criteria

The criteria for inclusion in this study were as follows: (1) Type of patients: adults (≥18 years) had a histologically proven diagnosis of malignant pleural mesothelioma with locally advanced or metastatic disease; (2) Type of study: phase II or phase III RCTs comparing antiangiogenic agents plus standard chemotherapy versus chemotherapy alone in first-line or second-line treatment; (3) There was at least one endpoint about OS or progression free survival (PFS) reported in the study; (4) If more than one publication showed data for the same study, only the most recent publication was included.

The criteria for exclusion were as follows: (1) Quasi-randomized trails which had a greater potential for bias were excluded. (2) Cross-over studies which had a significant impact on the overall effect of OS were also excluded.

Two reviewers (Wei Tian and Rui Wang) independently screened all the details of each study to confirm it met the inclusion criteria. When discrepancies arose, we resolved them through discussing with a third reviewer (Daidi Fu).

### Data extraction

Data were extracted from each included study by two reviewers (Wei Tian and Rui Wang) independently. Discrepancies and uncertainties were resolved by consensus with a third reviewer (Daidi Fu). OS was the primary endpoint, defined as the time from randomization to death from any cause. PFS was one of the secondary endpoints, defined as the time from randomization to disease progression or death. Other secondary endpoints included overall response rate (ORR) and serious toxicities. ORR was defined as the proportion of participants achieving confirmed complete response (CR) or partial response (PR). The hazard ratio (HR) and 95% confidence interval (CI) of time-to-event data including OS and PFS were synthesized and analyzed, as provided by Jayne F Tierney et al [14]. Risk ratio (RR) and 95% confidence interval of dichotomous data for ORR and toxicities were also analyzed. We extracted the HRs from the included studies or synthesized them from the reported events and the corresponding p-value of the log-rank statistics. When the above-mentioned data were not reported, we would digitize the Kaplan Meier curve and use Guyot’s method to calculate the summary estimate [15]. To assess the methodological quality of the 5 included studies, Jadad score method was adopted. The Jadad score, which ranged from 0 to 5, was based on three items, including descriptions of randomization, double blindness and withdrawals. When mentioning randomization, double blindness, and withdrawals, each study would be assigned a score of 1 point. If the appropriate methods of randomization or double blindness were described, an additional 1 point would be awarded. Studies scoring Jadad ≥ 3 were classified as high quality.

### Statistical analyses

Statistical analyses were performed using Review Manager 5.3 software. Time-to-event data were analyzed using HR. Dichotomous data were analyzed using RR. Statistical heterogeneity between studies was calculated by the chi-square test and expressed with the I^2^ index. For the I^2^ statistic, a I^2^ value higher than 50% meant great heterogeneity, 25%-50% meant moderate heterogeneity, less than 25% meant low heterogeneity. Fixed-effect model (Mantel-Haenszel method) was used if no significant heterogeneity was identified. In the absence of significant heterogeneity (p<0.1, or I^2^>50%), random-effect model or sensitivity analysis would be used. In addition, Egger’s test performed by stata 12.0 was also adopted to assess the possibility of publication bias. A two-sided p<0.05 was considered statistically significant.

## Results

### Study identification

**Fig 1:**
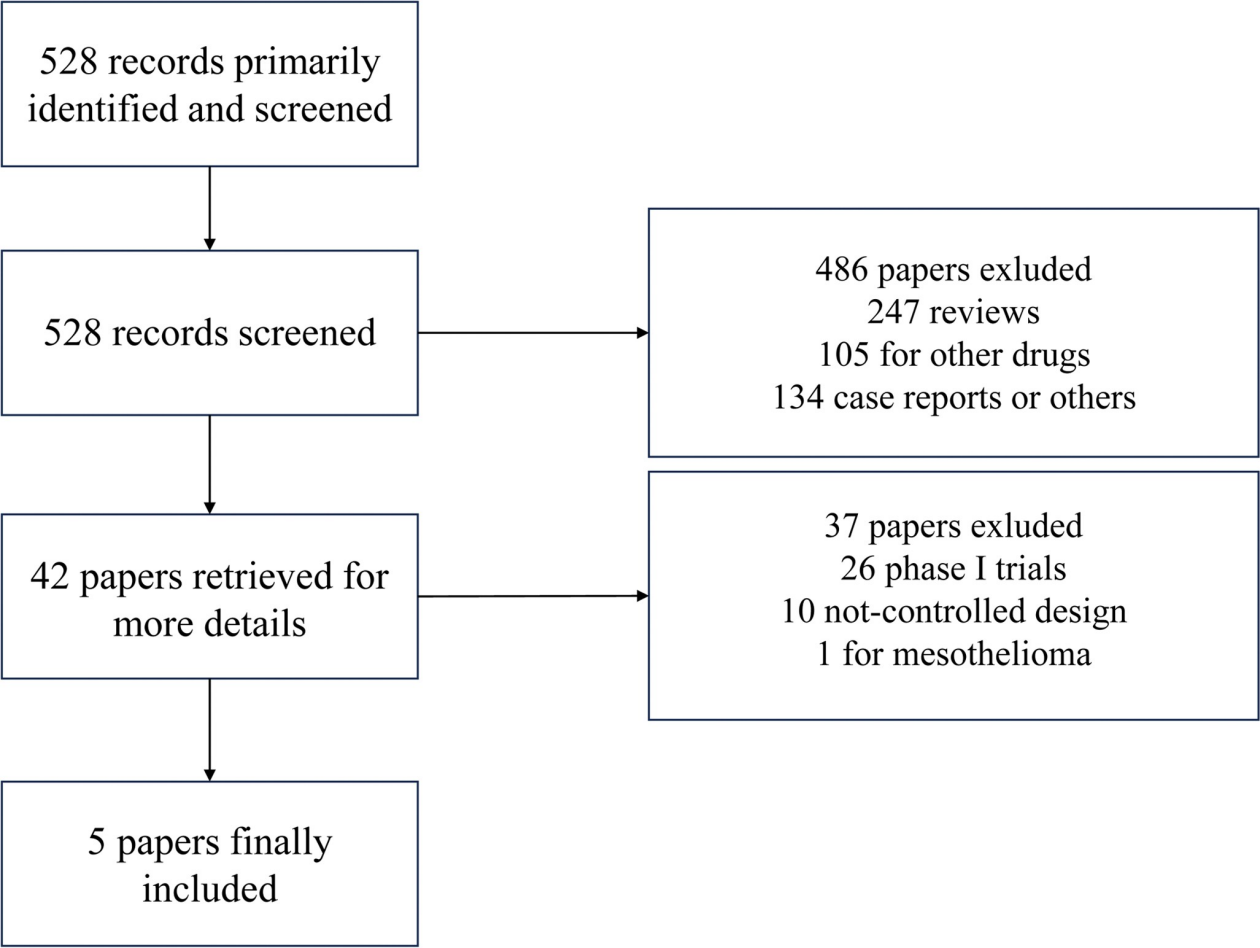
Flowchart for identification and inclusion of studies for this analysis.

528 records were rigorously screened in our search progress. Finally, 5 publications related to 5 RCTs (1250 patients) that compared the combination of antiangiogenic agents and chemotherapy with chemotherapy alone were included in the analysis [13, 16–19]. Three phase II [17–19] and two phase III [13, 16] trials were included. One trial reported the results of bevacizumab combined with chemotherapy [13]. Two trials compared nintedanib and chemotherapy with chemotherapy alone [16, 19]. One trial was designed to illustrate the efficacy of the addition of cediranib to chemotherapy [17]. One trial was about ramucirumab combined with chemotherapy [18]. **Fig 1** showed a flowchart for identifying relevant studies. The antiangiogenic mechanisms of above agents were described in **Table 2**.

**Table 2:** Antiangiogenic mechanisms of different agents.

antiangiogenic agents	antiangiogenic mechanisms
bevacizumab	monoclonal antibody inhibitor of VEGF
nintedanib	angiokinase inhibitor of VEGFR 1–3, PDGFR-α and β, FGFR 1–3
cediranib	tyrosine kinase inhibitor of VEGFR and PDGFR
ramucirumab	monoclonal antibody inhibitor of VEGFR 2

VEGF: vascular endothelial growth factor. VEGFR: vascular endothelial growth factor receptor. PDGFR: platelet-derived growth factor receptor. FGFR: fibroblast growth factor receptor.

### Characteristics of included studies

**Table 3** presented an overview of the patients’ baseline characteristics in the 5 studies. The included studies showed a relatively uniform distribution of patients’ characteristics at baseline. Treatment regimens and endpoints for the studies were showed in **Table 4**. **Table 5** provided methodological information that could potentially cause bias. Only 1 study included in this analysis was open-label [13]. The allocation concealment of randomization was presented in 2 studies [16, 18]. All the 5 included studies mentioned withdraw descriptions. It was important to note that there were differences in the exclusion criteria among the 5 studies. 2 studies excluded patients with substantial cardiovascular comorbidity [13, 17], while the other 3 studies did not [16, 18, 19].

**Table 3:** Patients’ characteristics of different arms in the included studies.

References	Histology	Treatment	Epithelioid	Patients	Male	ECOG ≤1 or	Median age
		Arms	(%)	Enrolled	(%)	KPS ≥70 (%)	(years)
Gerard et al. 2016 [13]	malignant pleural mesothelioma	combination	80	223	75	97	65.7
		chemotherapy	81	225	76	96	65.6
Federica et al. 2017 [19]	malignant pleural mesothelioma	combination	89	44	77	100	68
		chemotherapy	88	43	81	100	66
Anne S et al. 2019 [17]	malignant pleural mesothelioma	combination	76	45	84	93	72
		chemotherapy	74	47	85	94	72
Giorgio et al. 2019 [16]	malignant pleural mesothelioma	combination	96	229	72	100	66
		chemotherapy	97	229	74	100	66
Carmine et al. 2021 [18]	malignant pleural mesothelioma	combination	85	82	74	99	69
		chemotherapy	86	83	74	99	69

ECOG: Eastern Cooperative Oncology Group. KPS: Karnofsky Performance Status.

**Table 4:** Regimens and endpoints of included studies.

References	Regimens (per arm)	Interventions	Primary endpoint	Line
Gerard et al. 2016 [13]	Pem+CDDP+Bev	Arm A: Pem 500 mg/m^2^ iv d1, CDDP 75 mg/m^2^ iv d1, Bev 15 mg/kg iv d1, q3w.	OS	First
	Pem+CDDP	Arm B: Pem 500 mg/m^2^ iv d1, CDDP 75 mg/m^2^ iv d1, q3w.		
Federica et al. 2017 [19]	Pem+CDDP+Nin	Arm A: Pem 500 mg/m^2^ iv d1, CDDP 75 mg/m^2^ iv d1, Nin 400 mg/d po d2-21, q3w.	PFS	First
	Pem+CDDP	Arm B: Pem 500 mg/m^2^ iv d1, CDDP 75 mg/m^2^ iv d1, q3w.		
Anne S et al. 2019 [17]	Pem+CDDP+Ced	Arm A: Pem 500 mg/m^2^ iv d1, CDDP 75 mg/m^2^ iv d1, Ced 20 mg/d po d1-21, q3w.	PFS	First
	Pem+CDDP	Arm B: Pem 500 mg/m^2^ iv d1, CDDP 75 mg/m^2^ iv d1, q3w.		
Giorgio et al. 2019 [16]	Pem+CDDP+Nin	Arm A: Pem 500 mg/m^2^ iv d1, CDDP 75 mg/m^2^ iv d1, Nin 400 mg/d po d2-21, q3w.	PFS	First
	Pem+CDDP	Arm B: Pem 500 mg/m^2^ iv d1, CDDP 75 mg/m^2^ iv d1, q3w.		
Carmine et al. 2021 [18]	Gem+Ram	Arm A: Gem 1000mg/m^2^ iv d1 and d8, Ram 10mg/kg iv d1, q3w.	OS	Second
	Gem	Arm B: Gem 1000mg/m^2^ iv d1 and d8, q3w.		

Pem: pemetrexed. CDDP: cisplatin. Bev: bevacizumab. Nin: nintedanib. Ced: cediranib. Gem: gemcitabine. Ram: ramucirumab. OS: overall survival. PFS: progression free survival.

**Table 5:** Methodological details which might cause bias of included studies.

References	Phase	Random	Blind	Randomization description	Concealment description	Withdraw description	ITT analysis	Multicenter	Jadad score
Gerard et al. 2016 [13]	III	Yes	No	Yes	No	Yes	Yes	Yes	3
Federica et al. 2017 [19]	II	Yes	Yes	NC	NC	Yes	Yes	Yes	3
Anne S et al. 2019 [17]	II	Yes	Yes	NC	NC	Yes	Yes	No	3
Giorgio et al. 2019 [16]	III	Yes	Yes	Yes	Yes	Yes	Yes	Yes	5
Carmine et al. 2021 [18]	III	Yes	Yes	Yes	Yes	Yes	Yes	Yes	5

NC: no clear. ITT: intend-to-treat.

The methods of measurements to review tumor responses were not uniform between the included studies in our analysis. Due to the non-radial growth pattern and inconsistent treatment response, it was difficult to measure MPM radiographically. Inconsistent methods of measurements would influence the final results of PFS and ORR in the clinical trials. The criteria of measurement methods for MPM in the trials were either the RECIST (Response Evaluation Criteria In Solid Tumours) or the newer modified RECIST (mRECIST). The RECIST criteria for MPM was to measure the long axis of the tumor to achieve the longest uni-dimensional measurement. The 2 longest diameters of the pleural tumor were used to calculate the total measurement. The mRECIST criteria was to measure tumor thickness perpendicular to the chest wall or mediastinum in two positions at three separate levels. The MPM measure was the sum of the six measurements [20]. Only 1 study used the RECIST criteria to evaluate tumor response [18]. Other 3 studies used the mRECIST criteria [13, 16, 19]. Tumor measurements were determined by both RECIST and mRECIST in the study conducted by Anne et al [17]. Tumor response assessment was performed using computed tomography (CT) in 4 studies [13, 17–19]. Additionally, Giorgio et al conducted their study using either CT or magnetic resonance imaging (MRI) [16].

OS was defined as the duration from randomization to death from any cause. PFS was defined as the duration from randomization to disease progression or death from any cause. Two studies did not provide the detailed explanations of OS and PFS [16, 17]. The frequency of imaging test to assess tumor response varied among the included studies. Imaging scans were performed every 6 weeks in 3 studies [16, 17, 19] and every 9 weeks in 2 studies [13, 18]. Only 1 study explicitly stated that the imaging tests had been reviewed by an independent review committee [16].

### Overall survival

**Fig 2:**
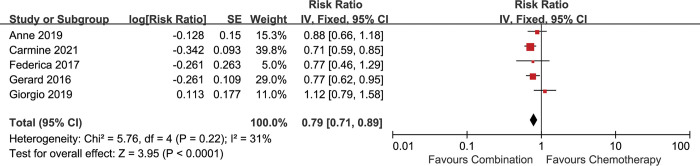
Comparison of OS between addition of antiangiogenic agents to chemotherapy and chemotherapy alone. SE: standard error. CI: confidence interval. IV: inverse variance.

The impact of antiangiogenic agents on OS was extracted directly from the 5 included studies. In the analysis, the combination of antiangiogenic agents and chemotherapy was associated with longer OS compared with chemotherapy alone [HR 0.79 (0.71–0.89), p<0.0001] **(Fig 2)**, without significant heterogeneity between studies (I^2^ = 31%, p = 0.22).

**Fig 3:**
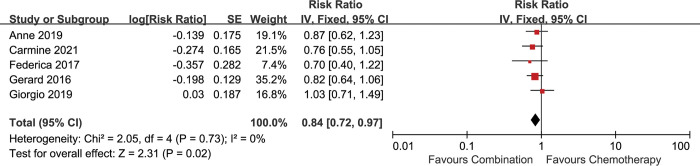
Comparison of OS between addition of antiangiogenic agents to chemotherapy and chemotherapy alone in the epithelioid subgroup. SE: standard error. CI: confidence interval. IV: inverse variance.

**Fig 4:**
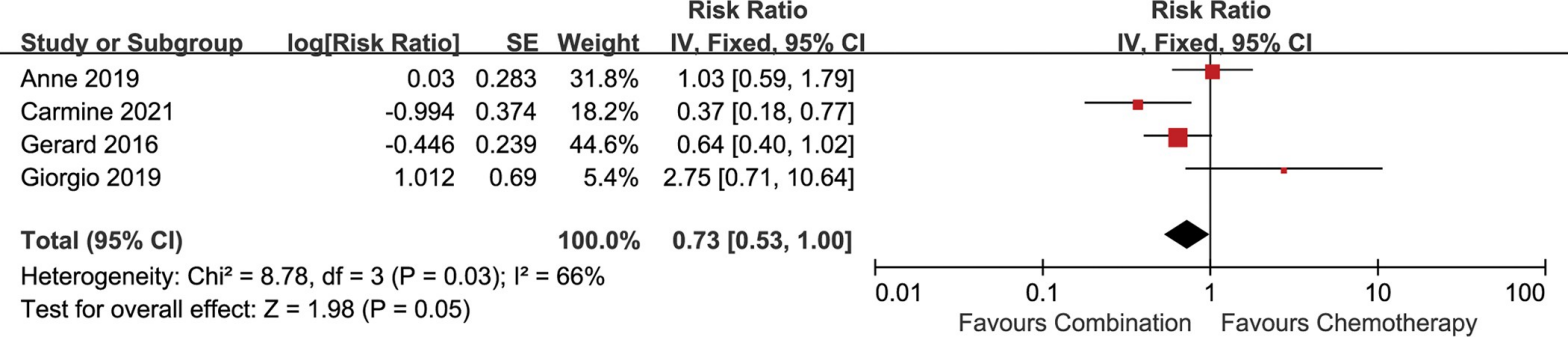
Comparison of OS between addition of antiangiogenic agents to chemotherapy and chemotherapy alone in the non-epithelioid subgroup. SE: standard error. CI: confidence interval. IV: inverse variance.

The patients in the 5 included studies were divided into epithelioid and non-epithelioid subgroups based on the pathological types. All the 5 included studies reported the data for epithelioid subgroup. 4 included studies reported available data for non-epithelioid subgroup [13, 16–18]. The analysis showed that the combination therapy was associated with longer survival in the epithelioid subgroup [HR 0.84 (0.72–0.97), p = 0.02] (I^2^ = 0%, p = 0.73) **(Fig 3)**, but not in the non-epithelioid subgroup [HR 0.73 (0.53–1.00), p = 0.05] (I^2^ = 66%, p = 0.03) **(Fig 4)**. Significant heterogeneity was found in the non-epithelioid subgroup analysis. When random-effect model was applied, no advantage on OS in the non-epithelioid subgroup was observed [HR 0.78 (0.43–1.40), p = 0.40] **(S1 Fig)**.

**Fig 5:**
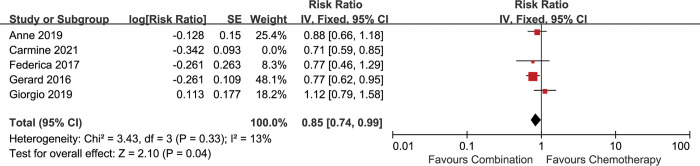
Comparison of OS between addition of antiangiogenic agents to chemotherapy and chemotherapy alone in the first-line setting. SE: standard error. CI: confidence interval. IV: inverse variance.

Of the 5 studies, 4 studies included patients that had not received any previous systematic therapies, and only the study conducted by Carmine et al included patients that had undergone first-line treatment. The superior OS advantage was observed in the first-line setting [HR 0.85 (0.74–0.99), p = 0.04], without significant heterogeneity (I^2^ = 13%, p = 0.33) **(Fig 5)**.

### Progression free survival

**Fig 6:**
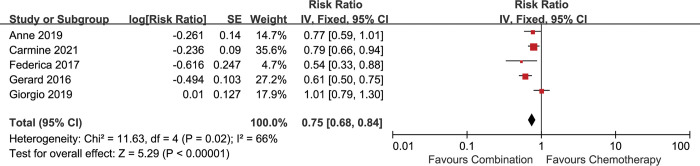
Comparison of PFS between addition of antiangiogenic agents to chemotherapy and chemotherapy alone. SE: standard error. CI: confidence interval. IV: inverse variance.

All the 5 included studies reported the data of PFS. 4 studies analyzed PFS according to the mRECIST criteria [13, 16, 17, 19], while the study conducted by Carmine et al used the RECIST criteria [18]. The addition of antiangiogenic agents to chemotherapy improved PFS compared with chemotherapy alone [HR 0.75 (0.68–0.84), p<0.00001], with significant heterogeneity among studies (I^2^ = 66%, p = 0.02) **(Fig 6)**. When random-effect model was applied, the advantage on PFS was also observed [HR 0.75 (0.62–0.91), p = 0.003] **(S2 Fig)**.

**Fig 7:**
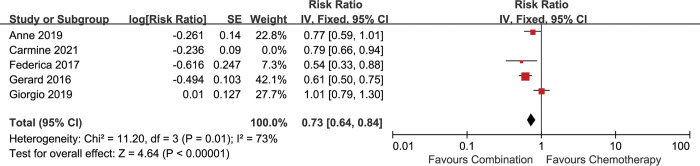
Comparison of PFS between addition of antiangiogenic agents to chemotherapy and chemotherapy alone according to the mRECIST criteria. SE: standard error. CI: confidence interval. IV: inverse variance.

To ensure consistency in the criteria for evaluating efficacy, we performed analysis of PFS by excluding the study conducted by Carmine et al. The analysis showed that addition of antiangiogenic agents was still associated with superior PFS [HR 0.73 (0.64–0.84), p<0.00001], with significant heterogeneity (I^2^ = 73%, p = 0.01) **(Fig 7)**. The superiority of PFS was also observed when random-effect model was used [HR 0.73 (0.56–0.96), p = 0.02] **(S3 Fig)**. The subgroup analysis of PFS in first-line setting was the same as the above result when excluding the study conducted by Carmine et al.

### Overall response rate

**Fig 8:**
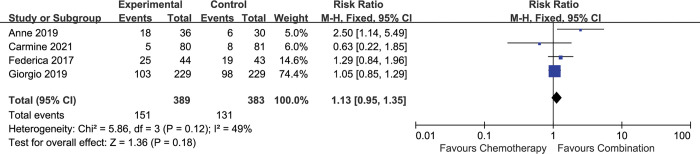
Comparison of ORR between addition of antiangiogenic agents to chemotherapy and chemotherapy alone. CI: confidence interval. M-H: Mantel-Haenszel.

4 included studies described the impact of combination therapy on ORR [16, 17–19]. The analysis showed no significant difference between two arms [HR 1.13 (0.95–1.35), p = 0.18], with moderate heterogeneity (I^2^ = 49%, p = 0.12) **(Fig 8)**.

**Fig 9:**
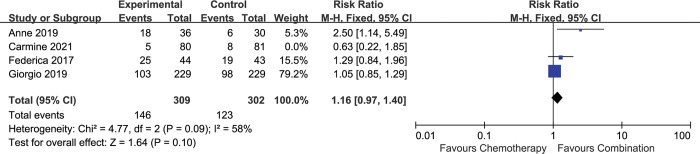
Comparison of ORR between addition of antiangiogenic agents to chemotherapy and chemotherapy alone in the first-line setting. CI: confidence interval. M-H: Mantel-Haenszel.

Subgroup analysis showed that no advantage of ORR was observed for antiangiogenic agents combined with chemotherapy in the first-line setting [HR 1.16 (0.97–1.40), p = 0.10], with significant heterogeneity (I^2^ = 58%, p = 0.09) **(Fig 9)**. When applying the random-effect model, no ORR advantage was observed [HR 1.30 (0.89–1.89), p = 0.17] **(S4 Fig)**.

### Toxicities

**Fig 10:**
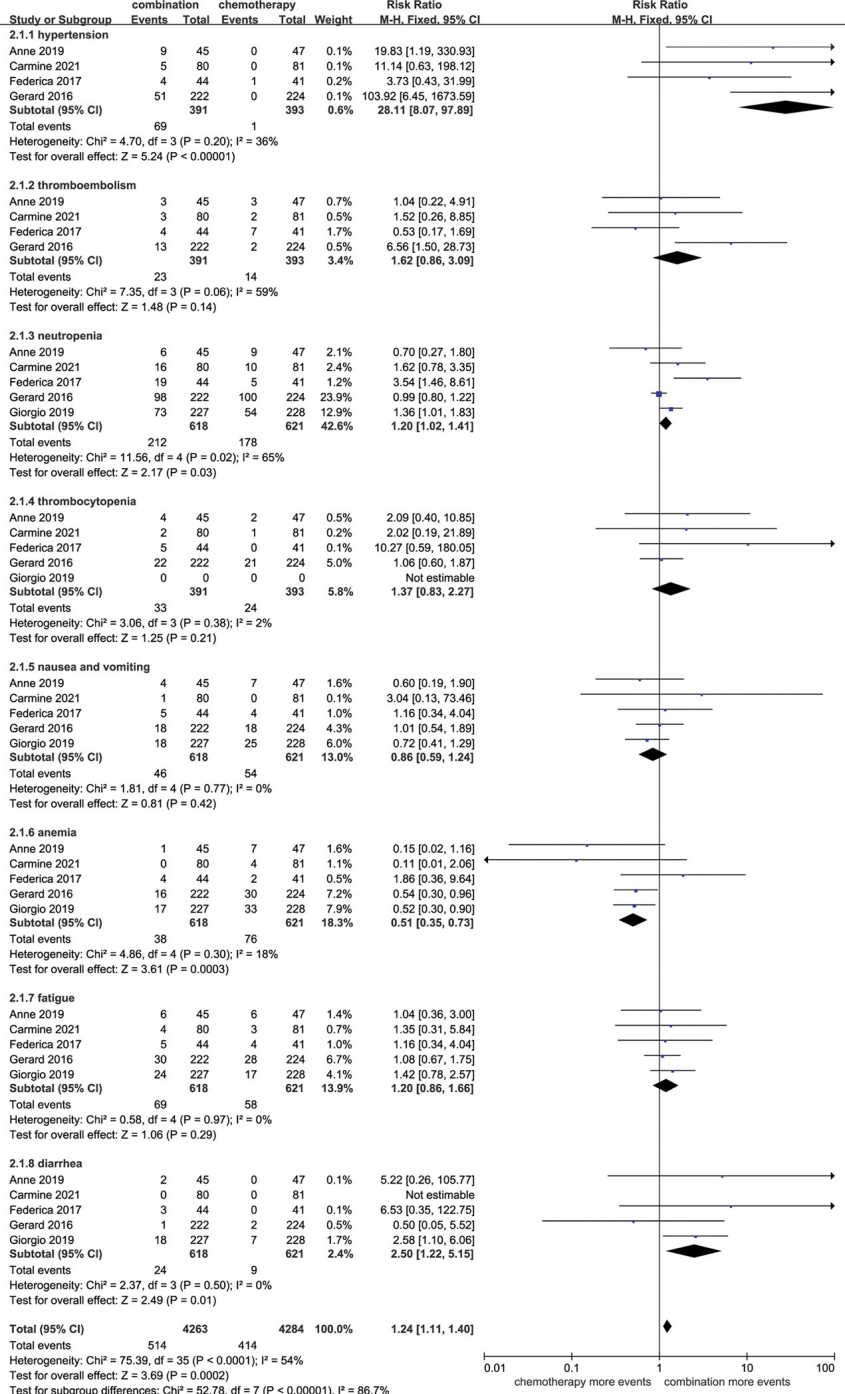
Comparison of toxicities between addition of antiangiogenic agents to chemotherapy and chemotherapy alone. CI: confidence interval. M-H: Mantel-Haenszel.

Reported toxicities with grade≥3 for the combination therapy were assessed in our analysis. We evaluated the toxicities of hypertension, thromboembolism, proteinuria and bleeding events primarily caused by antiangiogenic agents, and other common toxicities associated with conventional chemotherapy. Only 1 study reported the toxicity of proteinuria, including 1 case [18]. Bleeding events were rare, occurring in only 3 patients receiving combination therapy in 2 studies [13, 17]. There were more incidences of grade≥3 hypertension [RR 28.11 (8.07–97.89), p<0.00001], neutropenia [RR 1.20 (1.02–1.41), p = 0.03] and diarrhea [RR 2.50 (1.22–5.15), p = 0.01] when combining antiangiogenic agents with chemotherapy. Interestingly, the addition the antiangiogenic agents to chemotherapy reduced the incidence of grade≥3 anemia [RR 0.51 (0.35–0.73), p = 0.0003]. Equivalent frequencies of thromboembolism, thrombocytopenia, fatigue, nausea and vomiting were found between the two arms **(Fig 10)**.

### Publication bias

**Fig 11:**
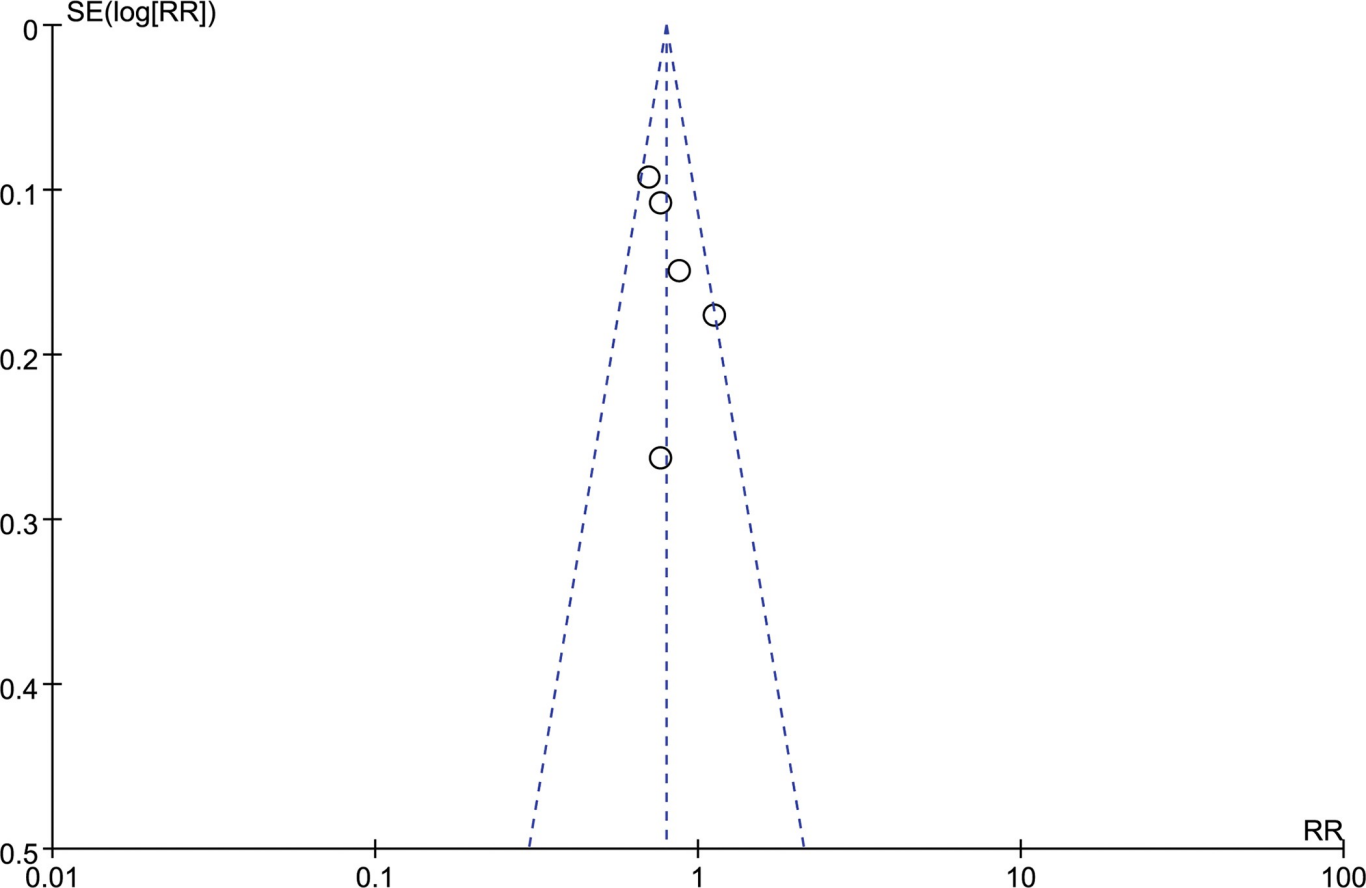
Funnel plot for evidence of publication bias.

We utilized a highly sensitive search strategy to select relevant studies, thereby minimizing the possibility of publication bias. And the collection of papers was strictly in accordance with the inclusion criteria. Publication bias was determined using funnel plots. Egger’s test provided statistical evidence for the symmetry of the funnel plot. There was no apparent publication bias detected in our analysis **(Fig 11)**. Egger’s test found no apparent evidence of bias in OS (p = 0.27) and PFS (p = 0.78).

## Discussion

Regimen of pemetrexed plus cisplatin, approved by Food and Drug Administration (FDA), had been considered as the standard care for first-line treatment in unresectable MPM. It had been proven to be quite challenging in making significant improvements in systemic therapy for advanced MPM. Antiangiogenic agents had been combined with the current standard of care in the first or second-line setting. However, the approach had yielded mixed results. Our analysis showed that addition of antiangiogenic agents to chemotherapy led to improvements on OS and PFS, with a tolerable toxicity profile.

Biomarkers that could reliably predict treatment response were of high clinical relevance. Increased platelet count could secrete angiogenic factors such as VEGF and PDGF, or directly stimulate the progression of angiogenesis to augment tumor growth in many types of solid tumors [21]. Higher platelet count had been considered to be a well-established predictive factor with poor OS and shorter PFS in MPM [22]. Furthermore, the increased expressions of VEGF and VEGFR in serum and tissues were associated with significantly poorer prognoses in patients with MPM [23]. Subgroup analyses according to baseline platelet count were only reported in two included studies in our analysis. As a result, no predictive biomarker that could guide treatment and improve patient selection was identified in our analysis. Additional studies should be warranted in future to identify the potential biomarkers, such as platelet count or VEGF concentration to inform treatment decision and patient selection.

In our analysis, no advantage on ORR was observed by adding antiangiogenic agents to chemotherapy in advanced MPM. Due to the complex morphology of MPM, response evaluation could easily cause high variability among investigators. Endpoints that should be assessed radiographically, such as PFS, ORR and duration of response, were challenging in trials for the pleural-based disease. Although mRECIST for mesothelioma was widely used in clinical trials, ability of the criteria to uniformly evaluate tumor response remained limited. The improvement on OS might be the preferred endpoint for definitive clinical trials in advanced MPM [24]. More studies should focus on improving the critical value of response classification and developing innovative alternative endpoints in this special pleural-based disease.

Several studies had reported the role of immunotherapy on MPM. Nivolumab monotherapy or nivolumab combined with ipilimumab both showed promising 12-week disease control rate (40% and 52%) in relapsed patients with MPM in the phase II randomized IFCT-1501 MAPS2 study [25]. The CheckMate 743 study was a phase III randomized controlled trial compared the efficacy of nivolumab plus ipilimumab with standard chemotherapy in patients with advanced MPM. Compared with chemotherapy, nivolumab plus ipilimumab enhanced OS (18.1 vs 14.1 months) in the first-line setting [26]. These studies indicated that immunotherapy could bring clinical benefits in patients with advanced MPM. Nivolumab and ipilimumab might be considered as the new standard of treatment in the near future. The combination of antiangiogenic agents and immune checkpoint inhibitors was considered to be synergistic in preclinical studies [27]. The IMPower 150 study was a phase III randomized controlled trial evaluated atezolizumab plus bevacizumab plus chemotherapy in patients with metastatic non-small-cell lung cancer. Compared with paclitaxel-carboplatin-bevacizumab, the addition of atezolizumab improved OS (19.2 months vs 14.7 months) [28]. More clinical studies should be needed to assess the addition of immune checkpoint inhibitors to antiangiogenics and chemotherapy in advanced MPM.

The following were some limitations existed in our analysis. Firstly, due to the small number of studies included, the power of the analysis was limited. Secondly, the combination of phase II and phase III trials was another limitation. OS was not always the primary endpoint in phase II trials. As a result, only two studies included in this analysis used OS as primary endpoint. The follow-up period in study using PFS as primary endpoint might be shorter than that using OS, which led to the non-equivalent data maturity between studies. Thirdly, clinical benefits of combining antiangiogenic agents and chemotherapy might be distinct in patients with different ages and general conditions. More subgroup analyses would be needed to make the conclusion stronger. But the dividing points of ages and general conditions for subgroup analyses were not uniform between studies, which acted as obstacles for further exploration. Fourthly, it was worth noting that hypoxia had the potential to enhance aggressiveness in malignant mesothelioma cells, thereby influenced various biological and molecular aspects. Previous in vitro experiments had substantiated the fact that hypoxia could stimulate clonogenicity, migration, invasion, and drug resistance to cisplatin in malignant mesothelioma cells [29]. However, none of the 5 studies included in this analysis provided information regarding the number of patients who underwent oxygen therapy, which could potentially impact the final outcomes of OS and PFS. Fifthly, among the 5 studies included, specific statistics on the number of patients who underwent pleural biopsy (percutaneous puncture or thoracoscopic surgery) were not provided. Most patients underwent thoracoscopic surgery would receive pleurodesis, which could potentially impact the prognosis. Finally, 2 studies excluded patients with significant cardiovascular comorbidity, potentially resulting in a study population with a more favorable prognosis. However, it was worth noting that the contraindications for the use of antiangiogenic agents were not strictly adhered to in a real-life setting, which might have a significant impact on the outcome, potentially leading to increased mortality. These factors should be taken into account in future clinical trials.

In conclusion, the combination of antiangiogenic agents and chemotherapy showed advantage compared with chemotherapy alone in patients with advanced MPM. Future studies should focus on identifying the potential biomarkers to distinguish patients who would most likely benefit from the combination regimen. Furthermore, prospective trials should be warranted to determine the synergetic effect of adding immune checkpoint inhibitors to antiangiogenic agents and chemotherapy.

## Supporting information

S1 Appendix PRISMA flow diagram. (DOC)

S1 Table PRISMA checklist. (DOCX)

S1 Fig Comparison of OS between addition of antiangiogenic agents to chemotherapy and chemotherapy alone in the non-epithelioid subgroup using random-effect model. SE: standard error. CI: confidence interval. IV: inverse variance.
(TIF)

S2 Fig Comparison of PFS between addition of antiangiogenic agents to chemotherapy and chemotherapy alone using random-effect model. SE: standard error. CI: confidence interval. IV: inverse variance.
(TIF)

S3 Fig Comparison of PFS between addition of antiangiogenic agents to chemotherapy and chemotherapy alone according to the mRECIST criteria using random-effect model. SE: standard error. CI: confidence interval. IV: inverse variance.
(TIF)

S4 Fig Comparison of ORR between addition of antiangiogenic agents to chemotherapy and chemotherapy alone in the first-line setting using random-effect model. CI: confidence interval. M-H: Mantel-Haenszel.
(TIF)

## References

[1] Sung H, Ferlay J, Siegel RL, Laversanne M, Soerjomataram I, Jemal A, et al. Global Cancer Statistics 2020: GLOBOCAN Estimates of Incidence and Mortality Worldwide for 36 Cancers in 185 Countries. CA Cancer J Clin. 2021; 71:209–49. doi: 10.3322/caac.21660 33538338

[2] Yap TA, Aerts JG, Popat S, Fennell DA. Novel insights into mesothelioma biology and implications for therapy. Nat Rev Cancer. 2017; 17:475–88. doi: 10.1038/nrc.2017.42 28740119

[3] Popat S, Baas P, Faivre-Finn C, Girard N, Nicholson AG, Nowak AK, et al. Malignant pleural mesothelioma: ESMO Clinical Practice Guidelines for diagnosis, treatment and follow-up(☆). Ann Oncol. 2022; 33:129–42. doi: 10.1016/j.annonc.2021.11.005 34861373

[4] Vogelzang NJ, Rusthoven JJ, Symanowski J, Denham C, Kaukel E, Ruffie P, et al. Phase III study of pemetrexed in combination with cisplatin versus cisplatin alone in patients with malignant pleural mesothelioma. J Clin Oncol. 2003; 21:2636–44. doi: 10.1200/JCO.2003.11.136 12860938

[5] Zhao Y, Adjei AA. Targeting Angiogenesis in Cancer Therapy: Moving Beyond Vascular Endothelial Growth Factor. Oncologist. 2015; 20:660–73. doi: 10.1634/theoncologist.2014-0465 26001391 PMC4571783

[6] Yasumitsu A, Tabata C, Tabata R, Hirayama N, Murakami A, Yamada S, et al. Clinical significance of serum vascular endothelial growth factor in malignant pleural mesothelioma. J Thorac Oncol. 2010; 5:479–83. doi: 10.1097/JTO.0b013e3181d2f008 20357617

[7] Kumar-Singh S, Weyler J, Martin MJ, Vermeulen PB, Van Marck E. Angiogenic cytokines in mesothelioma: a study of VEGF, FGF-1 and -2, and TGF beta expression. J Pathol. 1999; 189:72–8. 10.1002/(SICI)1096-9896(199909)189:1&lt;72::AID-PATH401&gt;3.0.CO;2-0 10451491

[8] Schelch K, Hoda MA, Klikovits T, Münzker J, Ghanim B, Wagner C, et al. Fibroblast growth factor receptor inhibition is active against mesothelioma and synergizes with radio- and chemotherapy. Am J Respir Crit Care Med. 2014; 190:763–72. doi: 10.1164/rccm.201404-0658OC 25188816

[9] Honda M, Kanno T, Fujita Y, Gotoh A, Nakano T, Nishizaki T. Mesothelioma cell proliferation through autocrine activation of PDGF-ββ receptor. Cell Physiol Biochem. 2012; 29:667–74. doi: 10.1159/000176386 22613967

[10] Garland LL, Chansky K, Wozniak AJ, Tsao AS, Gadgeel SM, Verschraegen CF, et al. Phase II study of cediranib in patients with malignant pleural mesothelioma: SWOG S0509. J Thorac Oncol. 2011; 6:1938–45. doi: 10.1097/JTO.0b013e318229586e 21964533 PMC3477852

[11] Papa S, Popat S, Shah R, Prevost AT, Lal R, McLennan B, et al. Phase 2 study of sorafenib in malignant mesothelioma previously treated with platinum-containing chemotherapy. J Thorac Oncol. 2013; 8:783–7. doi: 10.1097/JTO.0b013e31828c2b26 23571475

[12] Nowak AK, Millward MJ, Creaney J, Francis RJ, Dick IM, Hasani A, et al. A phase II study of intermittent sunitinib malate as second-line therapy in progressive malignant pleural mesothelioma. J Thorac Oncol. 2012; 7:1449–56. doi: 10.1097/JTO.0b013e31825f22ee 22895142

[13] Zalcman G, Mazieres J, Margery J, Greillier L, Audigier-Valette C, Moro-Sibilot D, et al. Bevacizumab for newly diagnosed pleural mesothelioma in the Mesothelioma Avastin Cisplatin Pemetrexed Study (MAPS): a randomised, controlled, open-label, phase 3 trial. Lancet. 2016; 387:1405–14. doi: 10.1016/S0140-6736(15)01238-6 26719230

[14] Tierney JF, Stewart LA, Ghersi D, Burdett S, Sydes MR. Practical methods for incorporating summary time-to-event data into meta-analysis. Trials. 2007; 8:16. doi: 10.1186/1745-6215-8-16 17555582 PMC1920534

[15] Guyot P, Ades AE, Ouwens MJ, Welton NJ. Enhanced secondary analysis of survival data: reconstructing the data from published Kaplan-Meier survival curves. BMC Med Res Methodol. 2012; 12:9. doi: 10.1186/1471-2288-12-9 22297116 PMC3313891

[16] Scagliotti GV, Gaafar R, Nowak AK, Nakano T, van Meerbeeck J, Popat S, et al. Nintedanib in combination with pemetrexed and cisplatin for chemotherapy-naive patients with advanced malignant pleural mesothelioma (LUME-Meso): a double-blind, randomised, placebo-controlled phase 3 trial. Lancet Respir Med. 2019; 7:569–80. doi: 10.1016/S2213-2600(19)30139-0 31103412

[17] Tsao AS, Miao J, Wistuba II, Vogelzang NJ, Heymach JV, Fossella FV, et al. Phase II Trial of Cediranib in Combination With Cisplatin and Pemetrexed in Chemotherapy-Naïve Patients With Unresectable Malignant Pleural Mesothelioma (SWOG S0905). J Clin Oncol. 2019; 37:2537–47. doi: 10.1200/JCO.19.00269 31386610 PMC7001793

[18] Pinto C, Zucali PA, Pagano M, Grosso F, Pasello G, Garassino MC, et al. Gemcitabine with or without ramucirumab as second-line treatment for malignant pleural mesothelioma (RAMES): a randomised, double-blind, placebo-controlled, phase 2 trial. Lancet Oncol. 2021; 22:1438–47. doi: 10.1016/S1470-2045(21)00404-6 34499874

[19] Grosso F, Steele N, Novello S, Nowak AK, Popat S, Greillier L, et al. Nintedanib Plus Pemetrexed/Cisplatin in Patients With Malignant Pleural Mesothelioma: Phase II Results From the Randomized, Placebo-Controlled LUME-Meso Trial. J Clin Oncol. 2017; 35:3591–600. doi: 10.1200/JCO.2017.72.9012 28892431

[20] Byrne MJ, Nowak AK. Modified RECIST criteria for assessment of response in malignant pleural mesothelioma. Ann Oncol. 2004; 15:257–60. doi: 10.1093/annonc/mdh059 14760119

[21] Buergy D, Wenz F, Groden C, Brockmann MA. Tumor-platelet interaction in solid tumors. Int J Cancer. 2012; 130:2747–60. doi: 10.1002/ijc.27441 22261860

[22] Pass HI, Giroux D, Kennedy C, Ruffini E, Cangir AK, Rice D, et al. Supplementary prognostic variables for pleural mesothelioma: a report from the IASLC staging committee. J Thorac Oncol. 2014; 9:856–64. doi: 10.1097/JTO.0000000000000181 24807157 PMC4132031

[23] Strizzi L, Catalano A, Vianale G, Orecchia S, Casalini A, Tassi G, et al. Vascular endothelial growth factor is an autocrine growth factor in human malignant mesothelioma. J Pathol. 2001; 193:468–75. doi: 10.1002/path.824 11276005

[24] Tsao AS, Lindwasser OW, Adjei AA, Adusumilli PS, Beyers ML, Blumenthal GM, et al. Current and Future Management of Malignant Mesothelioma: A Consensus Report from the National Cancer Institute Thoracic Malignancy Steering Committee, International Association for the Study of Lung Cancer, and Mesothelioma Applied Research Foundation. J Thorac Oncol. 2018; 13:1655–67. doi: 10.1016/j.jtho.2018.08.2036 30266660

[25] Scherpereel A, Mazieres J, Greillier L, Lantuejoul S, Dô P, Bylicki O, et al. Nivolumab or nivolumab plus ipilimumab in patients with relapsed malignant pleural mesothelioma (IFCT-1501 MAPS2): a multicentre, open-label, randomised, non-comparative, phase 2 trial. Lancet Oncol. 2019; 20:239–53. doi: 10.1016/S1470-2045(18)30765-4 30660609

[26] Baas P, Scherpereel A, Nowak AK, Fujimoto N, Peters S, Tsao AS, et al. First-line nivolumab plus ipilimumab in unresectable malignant pleural mesothelioma (CheckMate 743): a multicentre, randomised, open-label, phase 3 trial. Lancet. 2021; 397:375–86. doi: 10.1016/S0140-6736(20)32714-8 33485464

[27] Fukumura D, Kloepper J, Amoozgar Z, Duda DG, Jain RK. Enhancing cancer immunotherapy using antiangiogenics: opportunities and challenges. Nat Rev Clin Oncol. 2018; 15:325–40. doi: 10.1038/nrclinonc.2018.29 29508855 PMC5921900

[28] Socinski MA, Jotte RM, Cappuzzo F, Orlandi F, Stroyakovskiy D, Nogami N, et al. Atezolizumab for First-Line Treatment of Metastatic Nonsquamous NSCLC. N Engl J Med. 2018; 378:2288–301. doi: 10.1056/NEJMoa1716948 29863955

[29] Kim MC, Hwang SH, Kim NY, Lee HS, Ji S, Yang Y, et al. Hypoxia promotes acquisition of aggressive phenotypes in human malignant mesothelioma. BMC Cancer. 2018; 18:819. doi: 10.1186/s12885-018-4720-z 30111297 PMC6094475

