# Supplementary material for: Efficacy and safety profile of combining antiangiogenic agents with chemotherapy in patients with advanced malignant pleural mesothelioma: A systematic review and meta-analysis of randomized controlled trials
Source: PLoS One. 2023 Dec 21;18(12):e0295745. doi: 10.1371/journal.pone.0295745 (PMC10735007; doi:10.1371/journal.pone.0295745)
Supplement: S1 Appendix — (DOC) [file pone.0295745.s001.doc]

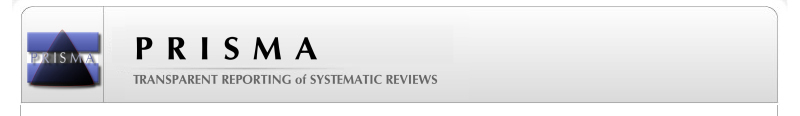
**PRISMA 2009 Flow Diagram**

**Screening**

**Included**

**Eligibility**

**Identification**

Records identified through database searching
(n = 594)

Additional records identified through other sources
(n = 0)

Records after duplicates removed
(n = 528)

Records screened
(n =528)

Records excluded
(n = 486)

247 reviews

105 for other drugs

134 case reports or others

Full-text articles assessed for eligibility
(n = 42)

Full-text articles excluded with reasons
(n = 37)

26 phase I trials

10 not-controlled design

1 for mesothelioma

Studies included in qualitative synthesis
(n = 5)

Studies included in quantitative synthesis (meta-analysis)
(n = 5)
