# Supplementary figures and images for: Efficacy and safety profile of combining antiangiogenic agents with chemotherapy in patients with advanced malignant pleural mesothelioma: A systematic review and meta-analysis of randomized controlled trials
Source: PLoS One. 2023 Dec 21;18(12):e0295745. doi: 10.1371/journal.pone.0295745 (PMC10735007; doi:10.1371/journal.pone.0295745)

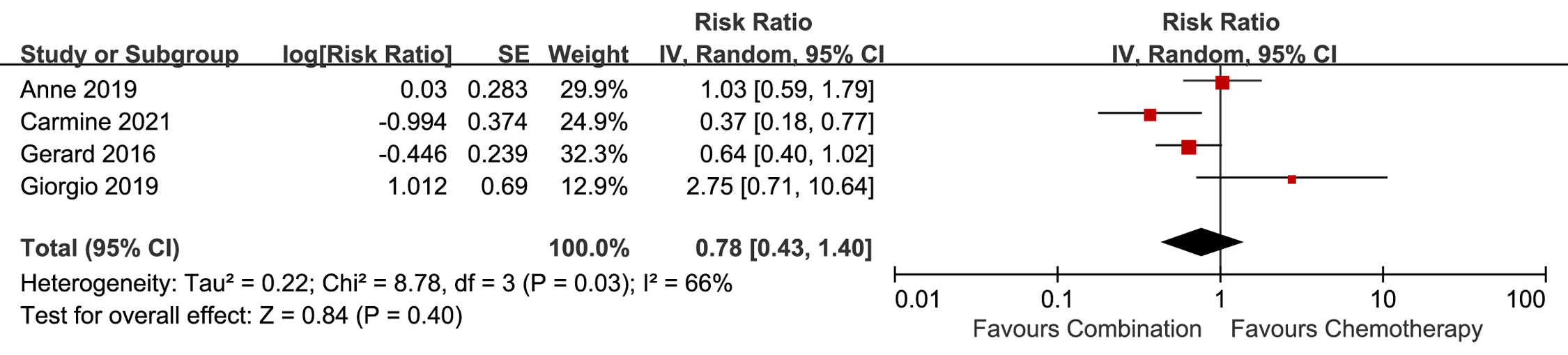

Supplement: S1 Fig — SE: standard error. CI: confidence interval. IV: inverse variance. (TIF) [file pone.0295745.s003.tif]

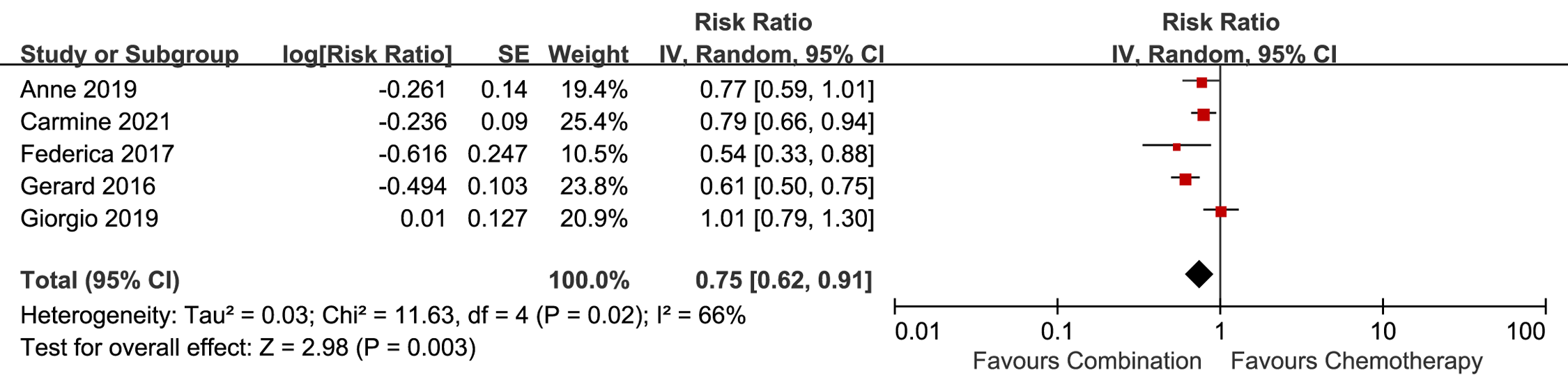

Supplement: S2 Fig — SE: standard error. CI: confidence interval. IV: inverse variance. (TIF) [file pone.0295745.s004.tif]

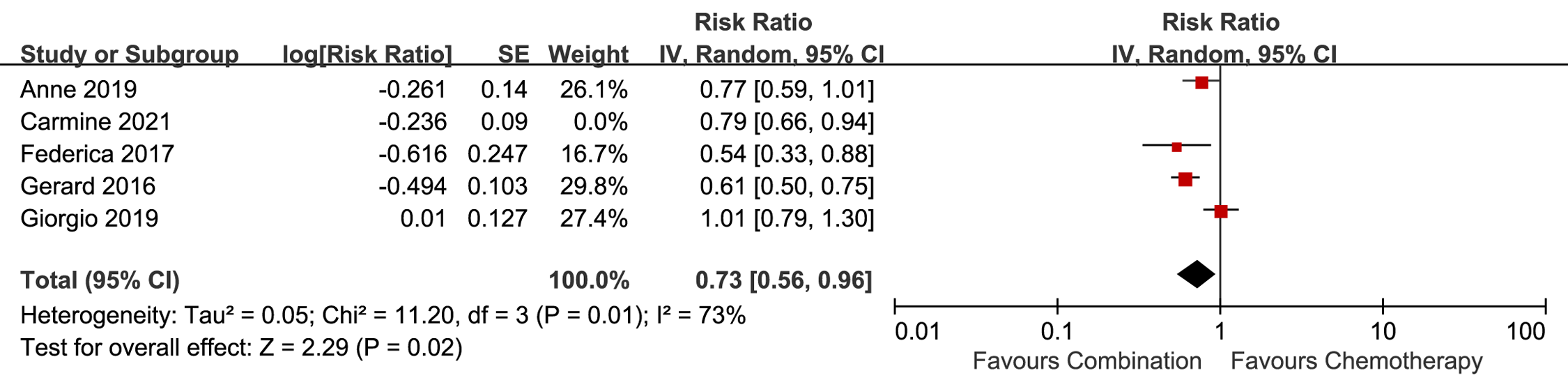

Supplement: S3 Fig — SE: standard error. CI: confidence interval. IV: inverse variance. (TIF) [file pone.0295745.s005.tif]

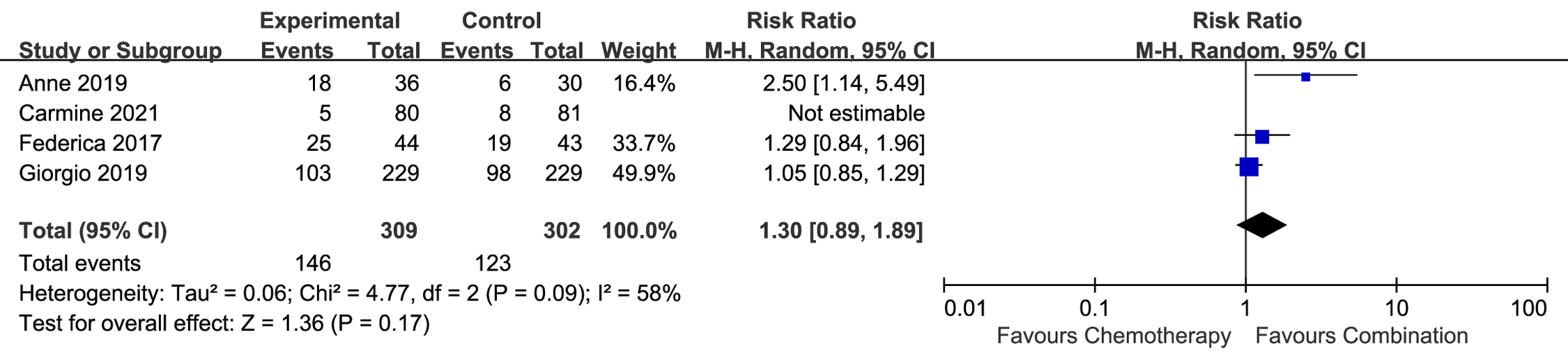

Supplement: S4 Fig — CI: confidence interval. M-H: Mantel-Haenszel. (TIF) [file pone.0295745.s006.tif]
