# Supplementary material for: Efficacy and safety profile of combining antiangiogenic agents with chemotherapy in patients with advanced malignant pleural mesothelioma: A systematic review and meta-analysis of randomized controlled trials
Source: PLoS One. 2023 Dec 21;18(12):e0295745. doi: 10.1371/journal.pone.0295745 (PMC10735007; doi:10.1371/journal.pone.0295745)
Supplement: Attachment — Submitted filename: Response to Reviewers.docx [file pone.0295745.s007.docx]

Reply for manuscript “Efficacy and safety profile of combining antiangiogenic agents with chemotherapy in patients with advanced malignant pleural mesothelioma: a systematic review and meta-analysis of randomized controlled trials”

 **Reply to editor:**

Dear editor:

Thank you for giving us the chance to submit the revised manuscript. We sincerely appreciate the reviewer’s constructive comments. Taking these valuable suggestions into consideration, we have made extensive revisions to the manuscript. This includes correcting errors and providing additional materials to improve its quality. Furthermore, we have ensured that the manuscript meets all of PLOS ONE's style requirements.

1. Please ensure that your manuscript meets PLOS ONE's style requirements, including those for file naming. The PLOS ONE style templates can be found at

https://journals.plos.org/plosone/s/file?id=wjVg/PLOSOne_formatting_sample_main_body.pdf and

https://journals.plos.org/plosone/s/file?id=ba62/PLOSOne_formatting_sample_title_authors_affiliations.pdf

Answer: We have carefully reviewed our manuscript to ensure that it meets PLOS ONE's style requirements.

2. Note from Emily Chenette, Editor in Chief of PLOS ONE, and Iain Hrynaszkiewicz, Director of Open Research Solutions at PLOS: Did you know that depositing data in a repository is associated with up to a 25% citation advantage (https://doi.org/10.1371/journal.pone.0230416)? If you’ve not already done so, consider depositing your raw data in a repository to ensure your work is read, appreciated and cited by the largest possible audience. You’ll also earn an Accessible Data icon on your published paper if you deposit your data in any participating repository (https://plos.org/open-science/open-data/#accessible-data).

Answer: We appreciate your kind reminder, but we have not made arrangements to deposit the raw data in a repository at this time.

3. We note that you have stated that you will provide repository information for your data at acceptance. Should your manuscript be accepted for publication, we will hold it until you provide the relevant accession numbers or DOIs necessary to access your data. If you wish to make changes to your Data Availability statement, please describe these changes in your cover letter and we will update your Data Availability statement to reflect the information you provide.

Answer: We revised the Data Availability Statement in the cover letter. All relevant data are within the paper and its Supporting information files.

4. Please include captions for your Supporting Information files at the end of your manuscript, and update any in-text citations to match accordingly. Please see our Supporting Information guidelines for more information: http://journals.plos.org/plosone/s/supporting-information.

Answer: We have included captions for Supporting Information at the end of our manuscript. Line 338-340.

5. Please review your reference list to ensure that it is complete and correct. If you have cited papers that have been retracted, please include the rationale for doing so in the manuscript text, or remove these references and replace them with relevant current references. Any changes to the reference list should be mentioned in the rebuttal letter that accompanies your revised manuscript. If you need to cite a retracted article, indicate the article’s retracted status in the References list and also include a citation and full reference for the retraction notice.

Answer: We have reviewed our reference list to ensure it meets the criteria outlined in the PLOS ONE guidelines for References. In response to the reviewer's question, we have included an additional paper [29] in the discussion section. Line 440-442.

**Reply to reviewer:**

**Dear reviewer:**

Thank you for dedicating your time and providing valuable feedback on our manuscript. We truly value your insightful comments, as they greatly contribute to the enhancement of our manuscript. We have thoroughly analyzed and addressed each comment individually, denoting the line number in the revised manuscript to track the alterations made. Our intention is to ensure that the revised version aligns more effectively with your expectations. If necessary, we are willing to make additional amendments to the manuscript to further enhance its quality.

**Reply to reviewer 1:**

Reviewer #1: The authors have done an incredible job in this research subject especially so since it is a rare malignancy and extensive efforts to identify bio markers have been unsuccessful.

I have a few questions for the authors though-

1)What kind of imaging modality was used in the patients? Was it a CECT or MRI?

Answer: In the revised manuscript, we included an illustration about the radiographical method used to assess tumor response in the 5 included studies. Line 178-180.

2)How many patients were hypoxic and on oxygen therapy since hypoxia itself is a tumor promoting state because it reduces apoptosis, increases tyrosine kinase activity, increases micro invasion and metastasis and all these could affect the outcome of the patients?

Answer: None of the 5 studies included in this analysis provided information regarding the number of patients who underwent oxygen therapy, which could potentially impact the final outcomes of OS and PFS. We have acknowledged this limitation in our manuscript. Line 315-320.

3)How many of the patients underwent a VATS as a diagnostic modality because VATS can cause pleurodesis in around 90% of times and do the authors think that this could affect the outcome of the study?

Answer: None of the 5 studies mentioned the number of patients underwent a VATS as a diagnostic modality. Specific statistics on the number of patients who underwent pleural biopsy (percutaneous puncture or thoracoscopic surgery) were not provided. This might have impacted the prognosis of patients. We added the illustration in the limitation part. Line 320-323.

4)Were patients with cardiovascular problems excluded from the study because that again could have a significant impact on the outcome in terms of increased mortality?

Answer: There were variations in the exclusion criteria among the 5 studies. 2 studies excluded patients with significant cardiovascular comorbidity, whereas the remaining 3 studies did not. We acknowledged this difference as a limitation in our manuscript. Line 323-327.
